# SLC39A8 Inhibits Ferroptosis by Regulating the Β-Catenin/TCF4/ GPX4 Signaling in Osteosarcoma

**DOI:** 10.2174/0115665240400149251029112234

**Published:** 2026-01-09

**Authors:** Siliang Tang, Guojun Tang, Liangyuan Chen, Mingjiang Luo, Jingshan Huang, Zihan Zhang, Shuo Wen, Zhihong Xiao

**Affiliations:** 1 Department of Spine Surgery, the Second Affiliated Hospital, Hengyang Medical School, University of South China, Hengyang, Hunan, 421001, China;; 2 Department of Union Hospital, West Hospital, Tongji Medical College, Huazhong University of Science and Technology, Wuhan, 430000, Hubei, China;; 3 Department of Spine Surgery, Lishui Hospital of Wenzhou Medical University, Lishui People's Hospital, Lishui, Zhejiang, 323050, China

**Keywords:** Osteosarcoma, SLC39A8, ferroptosis, β-catenin/TCF4 signaling, proliferation, migration

## Abstract

**Introduction:**

SLC39A8 has been implicated in various cancers; however, its specific role in osteosarcoma (OS) remains poorly understood. This study aims to elucidate the functional significance of SLC39A8 in OS progression.

**Methods:**

Using qRT-PCR and Western blot analysis, we analyzed SLC39A8 expression in osteosarcoma cells. Functional assays, including CCK-8, colony formation, and transwell assays, were employed to assess the impact of SLC39A8 on cell proliferation, migration, and invasion. Ferroptosis was evaluated by measuring lipid peroxidation, labile iron pool (LIP), Fe²⁺, malondialdehyde (MDA), reactive oxygen species (ROS), glutathione (GSH), and GPX4 expression.

**Results:**

Our results revealed that SLC39A8 is upregulated in osteosarcoma cells. The knockdown of SLC39A8 significantly suppressed cell proliferation, migration, and invasion while inducing ferroptosis, as evidenced by increased levels of LIP, Fe²⁺, MDA, and ROS, and decreased GSH and GPX4 expression. These effects were reversed by the ferroptosis inhibitor ferrostatin-1 (Fer-1). Furthermore, SLC39A8 overexpression activated the Wnt/β-catenin signaling pathway and upregulated GPX4 expression, effects that were abrogated by silencing β-catenin or TCF4. *In vivo* experiments confirmed that SLC39A8 knockdown inhibited tumor growth.

**Discussion:**

SLC39A8 is a key zinc and iron transporter. Studies have reported that SLC39A8 was significantly dysregulated in some cancers and was associated with their prognosis. SLC39A8 has been identified as an iron metabolism- and ferroptosis-related gene related to the prognosis of esophageal squamous cell carcinoma. Our study showed that SLC39A8 promotes osteosarcoma cell proliferation, migration, and invasion while suppressing ferroptosis by regulating β-catenin signaling. Our study further indicated that LF3 reversed SLC39A8-mediated ferroptosis in osteosarcoma cells by reducing GPX4 expression. Although our study shows that SLC39A8 regulates the β-catenin signaling pathway, the upstream regulatory mechanism remains to be investigated.

**Conclusion:**

Our findings demonstrate that SLC39A8 plays a pivotal role in osteosarcoma progression by modulating ferroptosis *via* the β-catenin/TCF4/GPX4 signaling pathways.

## 1 INTRODUCTION

Osteosarcoma (OS) is the most common primary bone cancer, with an incidence in children and adolescents of ~3–4.5 cases per million population per year [1, 2]. In the US, osteosarcoma represents under 1% of annual cancer diagnoses, with approximately 1,000 new cases identified yearly, about half of which occur in children and adolescents [2]. Despite advances in neoadjuvant chemotherapy and surgical techniques, the five-year survival rate for Osteosarcoma (OS) has reached nearly 70%; for patients with lung metastasis, it persists below 30% [3]. Therefore, identifying novel therapeutic targets and elucidating the underlying mechanisms driving OS progression is critical to improving patient outcomes.

Ferroptosis, a regulated form of cell death driven by iron-dependent lipid peroxidation, has emerged as a promising therapeutic target in cancer [4, 5]. Recent studies have highlighted the role of ferroptosis in tumor suppression, with evidence suggesting that pharmacological induction of ferroptosis can inhibit OS progression [6]. This has spurred interest in targeting ferroptosis as a potential strategy for OS treatment.

SLC39A8, a key zinc and iron transporter, has been implicated in multiple complex disorders [7]. Accumulating evidence indicates that SLC39A8 is significantly dysregulated in some cancers and is associated with their prognosis [8]. For instance, SLC39A8 has been implicated in a ferroptosis-related gene signature that predicts outcomes in colorectal cancer (CRC) patients [9], and its expression correlates negatively with the prognosis of esophageal squamous cell carcinoma (ESCC) patients [10]. Furthermore, SLC39A8 is identified as a key ZIP family gene in clear cell renal cell carcinoma (ccRCC), where its downregulation contributes to ccRCC progression [11].

SLC39A8 was closely associated with ferroptosis. Recent studies identified SLC39A8 as a key transporter driving ferroptosis in retinal pigment epithelium (RPE) cells [12]. Its mediated iron accumulation elevates lipid peroxidation, ultimately triggering RPE cell death. Another study demonstrated that targeting intracellular iron accumulation or inhibiting SLC39A8/SLC39A14 may mitigate iron-induced ferroptosis and impede vascular calcification progression [13]. Additionally, researchers have shown that ZIP8, encoded by SLC39A8, mediates coumestrol-induced apoptosis in CRC cells *via* the ferroptosis pathway [14]. However, the specific regulatory role and underlying mechanism of SLC39A8 in cancer ferroptosis remain largely undefined.

Wnt/β-catenin signaling has been reported to be a crucial carcinogenic pathway in the progression of osteosarcoma [15]. TCF4 is the most important transcription factor that acts as a switch of the Wnt/β-catenin pathway [16]. The activation of the Wnt/β-catenin signaling is associated with cancer ferroptosis [17, 18]. The relationship between SLC39A8, ferroptosis, and Wnt/β-catenin signaling in osteosarcoma remains uncertain.

In this study, we found that SLC39A8 acts as a potent ferroptosis inhibitor in OS and significantly promotes OS growth *in vitro* and *in vivo*.

Further data indicated that SLC39A8 up-regulated GPX4 through regulating β-catenin/TCF4 signaling. Our study provides insights into the potential therapeutic role of SLC39A8 in osteosarcoma.

## 2 MATERIALS AND METHODS

### 2.1 Cell Culture and Transfection

U2OS, MG63, and hFOB1.19 cells were purchased from the Cell Bank of the Chinese Academy of Sciences (Shanghai, China). U2OS, MG63 cells were cultured in DMEM medium supplemented with 10% fetal bovine serum (FBS, Invitrogen), hFOB1.19 cells were cultured in Ham’s F12/DMEM (Gibco) supplemented with 10% FBS. Cells were cultured in the incubator with 5% CO_2_ at 37℃.

sh-SLC39A8, si-β-catenin, and si-TCF4 were obtained from GenePharma. SLC39A8 and TCF4 overexpression plasmids were synthesized from RiboBio (Guangzhou, China).

Transfection was performed when OS cells were cultured to 80%-90% confluence. In detail, 2 µl Lipofectamine 2000 (Invitrogen, Waltham, USA) and siRNA or shRNA were added to 50 µl Opti-MEM (Gibco, NY, USA), respectively, and then mixed, followed by adding the above mixture to the cells for transfection for 24h.

For ferroptosis inhibition, OS cells (1×10^6^ cells) were treated with ferrostatin-1 (1 μM, Selleck, Houston, TX, USA) for 24 h.

### 2.2 Quantitative Real-time PCR (qRT-PCR)

Total RNAs were isolated from OS cells using TRIzol Reagent (Invitrogen, USA). The relative expression was normalized to GAPDH and calculated using the 2^−ΔΔCt^ method. The primer sequences were listed as follows: SLC39A8 (forward: 5′-TGGTTGCACCCCTCACAAAT-3′ and reverse: 5′-CACATGGTGCACTGAAACCG-3′); GPX4 (forward: 5′-GAAGCAGGAGCCAGGGAGT-3′ and reverse: 5′-ACGCAGCCGTTCTTGTCG-3′); GAPDH (forward: 5′-GATGCCCCCATGTTCGTCAT-3′ and reverse: 5′-CCATTCCCCAGCTCTCATACC-3′).

### 2.3 Cell Viability Assay

Two thousand cells were seeded and plated in a 96-well plate. The wells were added with 10 µL CCK-8 solution (Dojindo, Japan) and incubated for 2 h. Cell viability was assessed by detecting the absorbance at 450 nm.

### 2.4 Colony Formation Assay

Cells were cultured in 6-well plates (1000 cells/well) for two weeks. The cells were fixed with 4% paraformaldehyde and stained with 0.5% crystal violet solution. Cell colonies were counted.

### 2.5 Western Blotting

Total proteins were extracted using RIPA lysis buffer (Beyotime, China). The members were blocked using 5% non-fat milk and incubated with antibodies: SLC39A8 (1:000, Proteintech), GPX4 (1:1000, Cell Signaling Technology), β-catenin (1:1000, Abcam), TCF4 (1:1000, Abcam), Histone H3 (1:2000; Abcam), GAPDH (1:2000, Abcam) at 4°C overnight. The members were then incubated with the corresponding secondary antibodies. The bands were detected by electrochemiluminescence and quantified by densitometry.

### 2.6 Transwell Assay

Cells were suspended in DMEM and seeded in a transwell chamber (Corning, CA). Matrigel-coated membranes were utilized for the invasion assay, while uncoated membranes were used for the migration assay. 24h later, the cells on the lower surface of the membrane were fixed and stained with 0.5% crystal violet. Experiments were performed as previously described [19].

### 2.7 MDA, GSH, ROS, Labile Iron Pool (LIP), Fe2+ Assays

The level of MDA was measured using the Lipid Peroxidation MDA Assay Kit (Beyotime, China). The GSH level was assessed using a GSH and GSSG Assay Kit (Beyotime, S0053). ROS levels were measured by C11-BODIPY581/591 (GC40165, GLPBIO). LIP and Fe²⁺ levels were measured by an iron assay kit (Abcam).

### 2.8 Immunofluorescence

Seed cells on poly-L-lysine-coated coverslips in 24-well plates. Fix with 4% paraformaldehyde for 15 min at room temperature (RT). After fixation, the cells were washed with PBS and permeabilized using 0.2% Triton X-100 in PBS for 10 min at RT. Wash 3× with PBS (5 min each). The cells are incubated with blocking solution (0.1% Triton/PBS containing 5% BSA) for 1 hr (RT), followed by overnight incubation with specific antibodies. Wash 3×with PBS. Cells were incubated with fluorescent secondary antibodies for 2 hr at RT in the dark. Wash 3× with PBS (5 min each). Cells were incubated with DAPI (1 µg/mL in PBS) for 5 min (RT). Wash 2× with PBS. Visualize using a fluorescence microscope.

### 2.9 Xenograft Model

All animal experiments were performed in accordance with the standards outlined in the US National Research Council's Guide for the Care and Use of Laboratory Animals, and were approved by the Institutional Animal Care and Use Committee of the Second Affiliated Hospital, University of South China (No. 2023078). Female BALB/C nude mice weighing 18-20 g and aged 4-6 weeks were purchased from Shanghai SLAC Experimental Animal Co., Ltd. and raised without specific pathogens. Briefly, twelve female athymic nude mice were divided equally into two groups. U2OS cells (2×10^6^) transfected with sh-NC or sh-SLC39A8 were subcutaneously injected into the left flank of the nude mice. Tumor growth was monitored, and tumor volume was quantified using the following formula: Volume = (length × width^^2^)/2. 5 weeks later, the tumors were excised. The xenograft tissues were used for Ki67 immunohistochemistry.

### 2.10 Statistical Analysis

Data were represented as means ± standard deviation (SD). Statistical analysis was conducted using SPSS 21.0 (SPSS, Chicago, IL). Differences between the two groups were analyzed using Student’s t-test. One-way analysis of variance (ANOVA) followed by Tukey’s post-hoc test was used for comparisons among multiple groups. *P*<0.05 was considered statistically significant.

## 3 RESULTS

### 3.1 SLC39A8 is Up-regulated in Osteosarcoma

We examined SLC39A8 expression levels in osteosarcoma cells. qRT-PCR and Western blot assays demonstrated that SLC39A8 was highly expressed in osteosarcoma cells in comparison to hFOB1.19 cells (Figs. **1A**, **B**).

### 3.2 SLC39A8 Knockdown Inhibits Osteosarcoma Cell Proliferation, Migration, and Invasion

To investigate the role of SLC39A8 in osteosarcoma cells, SLC39A8 was silenced by transfection of sh-SLC39A8 in MG-63 and U2OS cells. Knockdown efficiency was confirmed at both mRNA and protein levels (Fig. **2A**). Notably, the CCK-8 and colony formation assays revealed that silencing SLC39A8 markedly inhibited the proliferation of osteosarcoma cells (Figs. **2B**, **C**). Additionally, transwell assays demonstrated significant reductions in cell migration and invasion (Figs. **2D**, **E**), underscoring SLC39A8's role in promoting aggressive OS phenotypes.

### 3.3 SLC39A8 Knockdown Induces Osteosarcoma Cell Ferroptosis

Several studies reported that SLC39A8 is implicated in tumor ferroptosis [14, 20]. Therefore, we explored the relationship between SLC39A8 and ferroptosis, a form of regulated cell death implicated in the progression of osteosarcoma [21]. We treated SLC39A8-knockdown cells with the ferroptosis inhibitor ferrostatin-1(Fer-1). As we expected, the Fer-1 (1 μM) strikingly reversed the cell viability inhibited by SLC39A8-down-regulation (Fig. **3A**). Silencing SLC39A8 increased levels of iron, Fe²⁺, LIP, MDA, and lipid ROS (Figs. **3B**–**F**), while reducing GSH levels (Fig. **3G**). Additionally, GPX4 expression, a key regulator of ferroptosis, was significantly inhibited following SLC39A8 knockdown. (Fig. **3H**). These effects were reversed by the ferroptosis inhibitor Fer-1, which restored GSH and GPX4 levels and reduced ferroptosis markers (Figs. **3B**-**H**).

### 3.4 SLC39A8 Induces GPX4 Expression Through β-catenin/TCF4

We next investigated the underlying mechanisms through which SLC39A8 influences GPX4 expression and ferroptosis. The Wnt/β-catenin signaling pathway, known for its role in tumor progression and ferroptosis regulation [16], was a focal point of our study. The activation of Wnt/β-catenin signaling relies on the translocation of β-catenin into the nucleus. Our findings revealed that β-catenin levels increased in the nucleus of SLC39A8-overexpressing osteosarcoma cells (Fig. **4A**). Further analysis showed that SLC39A8 overexpression promoted the nuclear localization of β-catenin (Fig. **4B**). SLC39A8 overexpression enhanced GPX4 expression, which was abolished by β-catenin knockdown (Figs. **4C**, **D**). Previous studies have identified TCF4 as a key transcriptional effector in the Wnt/β-catenin pathway [16]. We further explored whether SLC39A8 induces GPX4 expression in conjunction with TCF4. Our results indicated that TCF4 overexpression increased both mRNA and protein levels of GPX4 (Figs. **4E**, **F**), whereas silencing TCF4 reduced GPX4 expression (Figs. **4G**, **H**). Moreover, SLC39A8 overexpression elevated TCF4 and GPX4 expression, and these effects were reversed by TCF4 knockdown (Figs. **4G**, **H**).

### 3.5 SLC39A8 Inhibits Ferroptosis in Osteosarcoma Cells *Via* Wnt/β-catenin Signaling

LF3 is a Wnt/β-catenin signaling inhibitor [21, 22]. To determine whether SLC39A8 inhibits ferroptosis through Wnt/β-catenin signaling, we treated SLC39A8-overexpressing OS cells with LF3. We observed that LF3 diminished the effects of SLC39A8 overexpression on cell viability (Fig. **5A**). LF3 reversed the inhibitory effects of SLC39A8 on ferroptosis, leading to increased levels of iron, Fe^2+^, LIP, MDA, and lipid ROS (Figs. **5B**-**F**), while reducing GSH levels (Fig. **5G**) and GPX4 expression (Fig. **5H**).

### 3.6 Silencing SLC39A8 Inhibits Tumor Growth *In vivo*

Finally, we evaluated the *in vivo* effects of SLC39A8 using xenograft mouse models. SLC39A8 knockdown significantly reduced tumor volume and weight (Figs. **6A**, **B**). Immunohistochemical analysis further confirmed a decrease in Ki-67 expression in the SLC39A8 knockdown group (Fig. **6C**), suggesting that targeting SLC39A8 could be a viable therapeutic strategy to inhibit osteosarcoma growth.

## 4 DISCUSSION

Osteosarcoma exhibits highly invasive and metastatic potential. Although neoadjuvant chemotherapy and surgical techniques have improved, metastatic osteosarcoma remains incurable. Therefore, it is crucial to elucidate the underlying mechanisms driving osteosarcoma progression and to develop effective treatment strategies. Our study demonstrates that SLC39A8 plays a critical role in osteosarcoma progression by promoting cell proliferation, migration, and invasion while suppressing ferroptosis through the β-catenin/TCF4 signaling pathway.

Ferroptosis, a recently identified form of regulated cell death, is characterized by distinct biochemical features, including iron accumulation, lipid peroxidation, reactive oxygen species (ROS) generation, and glutathione depletion [23, 24]. This unique cell death pathway has attracted increasing attention in cancer research due to its emerging role in inhibiting tumor growth [25], positioning it as a promising therapeutic target across various malignancies.

Notably, accumulating evidence suggests that ferroptosis plays a critical role in modulating osteosarcoma progression. For instance, studies have demonstrated that baicalin exerts anti-osteosarcoma effects through a mechanism dependent on Nrf2/xCT/ GPX4-mediated ferroptosis [26]. Additionally, Chen *et al*. reported that KDM4A reduces the sensitivity of osteosarcoma cells to cisplatin by inhibiting ferroptosis [27]. Li *et al*. reported that FANCD2 suppresses ferroptosis by regulating JAK2/STAT3 signaling in osteosarcoma [28].

SLC39A8, also known as ZIP8, is a membrane transporter that regulates IFN-γ expression in T cells and is closely associated with the progression of hepatocellular carcinoma [29, 30]. High SLC39A8 expression in patients with colon adenocarcinoma correlates with reduced overall survival [31]. SLC39A8 has been identified as an iron metabolism- and ferroptosis-related gene associated with the prognosis of esophageal squamous cell carcinoma [10]. Yang *et al*. reported that coumestrol promotes apoptosis in CRC cells by interacting with ZIP8 *via* the ferroptosis pathway [14]. SLC39A8 is a ferroptosis-related gene (FRG) that serves as a prognostic marker for pancreatic adenocarcinoma [20]. A recent study identified SLC39A8 as part of a ferroptosis-related gene signature linked to immunity and predictive of prognosis in patients with osteosarcoma [32]. However, these studies have not extensively investigated the molecular mechanisms underlying SLC39A8's role in tumor ferroptosis. Our study demonstrated that SLC39A8 expression was upregulated in osteosar-coma cells. Furthermore, SLC39A8 knockdown inhibited cell proliferation, migration, and invasion, induced ferroptosis, and suppressed tumor growth *in vivo*.

The activation of Wnt/β-catenin signaling is recognized as a mechanism that enables tumor cells to evade ferroptosis. For instance, DDX5 has been shown to enhance the anti-tumor effects of sorafenib by inhibiting Wnt/β-catenin activation, thereby promoting ferroptosis in hepatocellular carcinoma [17]. Recent studies indicate that USP8 inhibits ferroptosis by activating Wnt/β-catenin signaling in hepatocellular carcinoma [18]. Moreover, activation of Wnt/β-catenin signaling has been associated with increased GPX4 expression and ferroptosis resistance in gastric cancer [16]. Our findings indicate that SLC39A8 inhibits ferroptosis in osteosarcoma cells by activating β-catenin/TCF4/GPX4 signaling. LF3, a 4-thioureido-benzenesulfonamide derivative, has been reported to disrupt the interaction between β-catenin and TCF4 [16]. Our study demonstrated that LF3 reversed SLC39A8-mediated ferroptosis in osteosarcoma cells by reducing GPX4 expression. Therefore, targeting β-catenin/TCF4 using LF3 or lipid nanoparticle (LNP)-mediated siβ-catenin delivery to suppress Wnt/β-catenin activation represents a promising therapeutic approach in osteosarcoma treatment. Furthermore, developing an SLC39A8 inhibitor that induces ferroptosis in osteosarcoma cells may help reverse or stall osteosarcoma progression. However, this therapeutic strategy requires further investigation.

## 5 STUDY LIMITATIONS

It is important to acknowledge several limitations of our study. Firstly, the upstream regulatory mechanism by which SLC39A8 activates the Wnt/β-catenin pathway remains unclear. Secondly, our study did not include clinical samples to verify the expression and prognostic value of SLC39A8 in osteosarcoma patients. Further studies are needed to elucidate the mechanism by which SLC39A8 induces GPX4 expression and activates Wnt/β-catenin signaling，and to validate its clinical significance in patient cohorts.

## CONCLUSION

In conclusion, our study identifies SLC39A8 as a critical regulator of osteosarcoma progression and ferroptosis. By modulating the β-catenin/TCF4/GPX4 signaling pathway, SLC39A8 promotes tumor growth and inhibits ferroptosis. These findings improve our understanding of osteosarcoma biology and suggest potential therapeutic strategies targeting SLC39A8 and its associated pathways.

## AUTHORS’ CONTRIBUTIONS

The author's confirm their contribution to the paper as follows: ZX designed the study, supervised the data collection; ST, GT, LC, ML, and JH performed experiments; ZZ and SW analyzed the data; ST and ZX wrote the manuscript and reviewed the draft. All authors have read and approved the manuscript.

## Figures and Tables

**Fig. (1):**
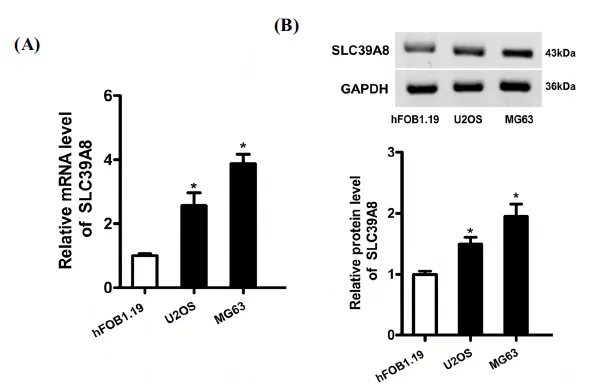
SLC39A8 was up-regulated in OS cells. (**A**, **B**) The expression of SLC39A8 was elevated in OS cell lines. Data are expressed as mean ± standard deviation. The experiments were repeated 3 times. * *P*<0.05 *vs.* FOB1.19.

**Fig. (2):**
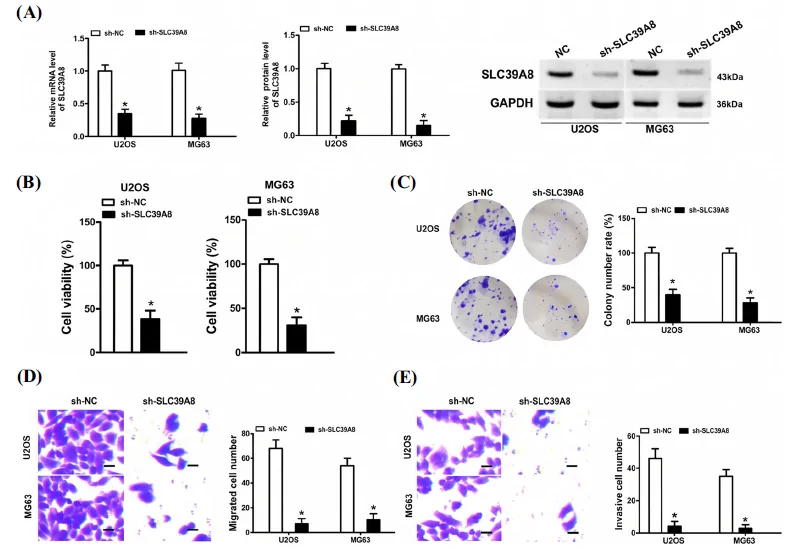
SLC39A8 silencing inhibits cell proliferation, migration, and invasion. (**A**) SLC39A8 expression was detected by RT-qPCR and western blot. (**B**) Cell viability was determined by CCK-8 assay. (**C**) Proliferation of OS cells was determined by colony formation assay. (**D**-**E**) OS cell migration and invasion were assessed by Transwell assay. Scale bar: 100 μm. Data are expressed as mean ± standard deviation. The experiments were repeated 3times. *P<0.05 *vs.* sh‑NC.

**Fig. (3):**
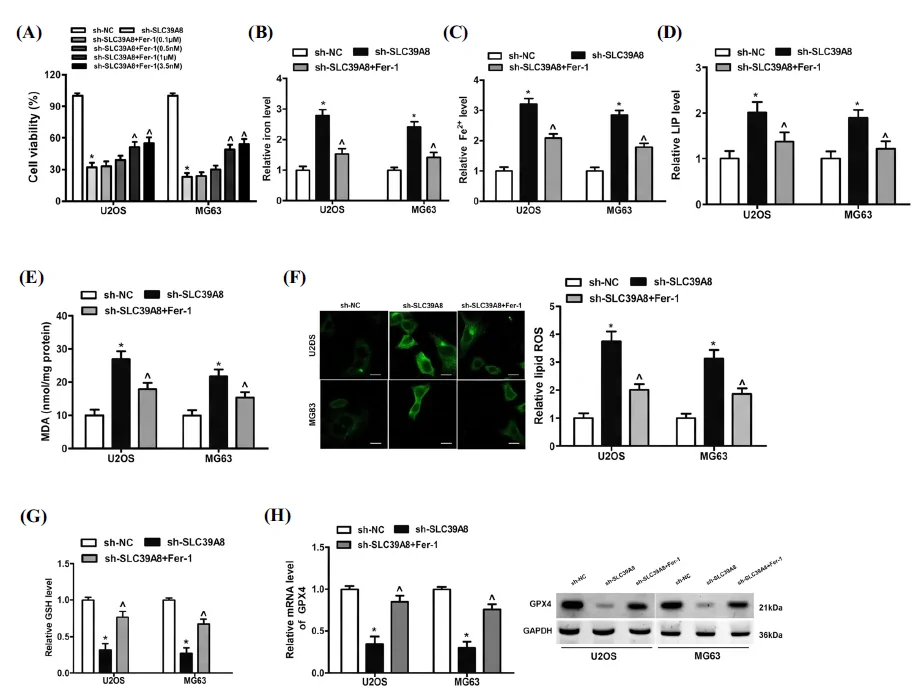
SLC39A8 knockdown promoted OS cell ferroptosis. (**A**) Cell viability was measured in sh-NC and sh-SLC39A8 OS cells treated with ferrostatin-1 (1 μM). (**B**-**D**) The levels of iron, ferrous iron, and LIP were determined. (**E**-**G**) The levels of MDA, lipid ROS levels, and GSH were measured. Scale bar: 10 μm. (H) The mRNA and protein level of GPX4 was detected by qRT-PCR and western blot. Data are expressed as mean ± standard deviation. The experiments were repeated 3 times. *P <0.05 *vs.* sh‑NC; ^*P*<0.05 *vs.* sh-SLC39A8.

**Fig. (4):**
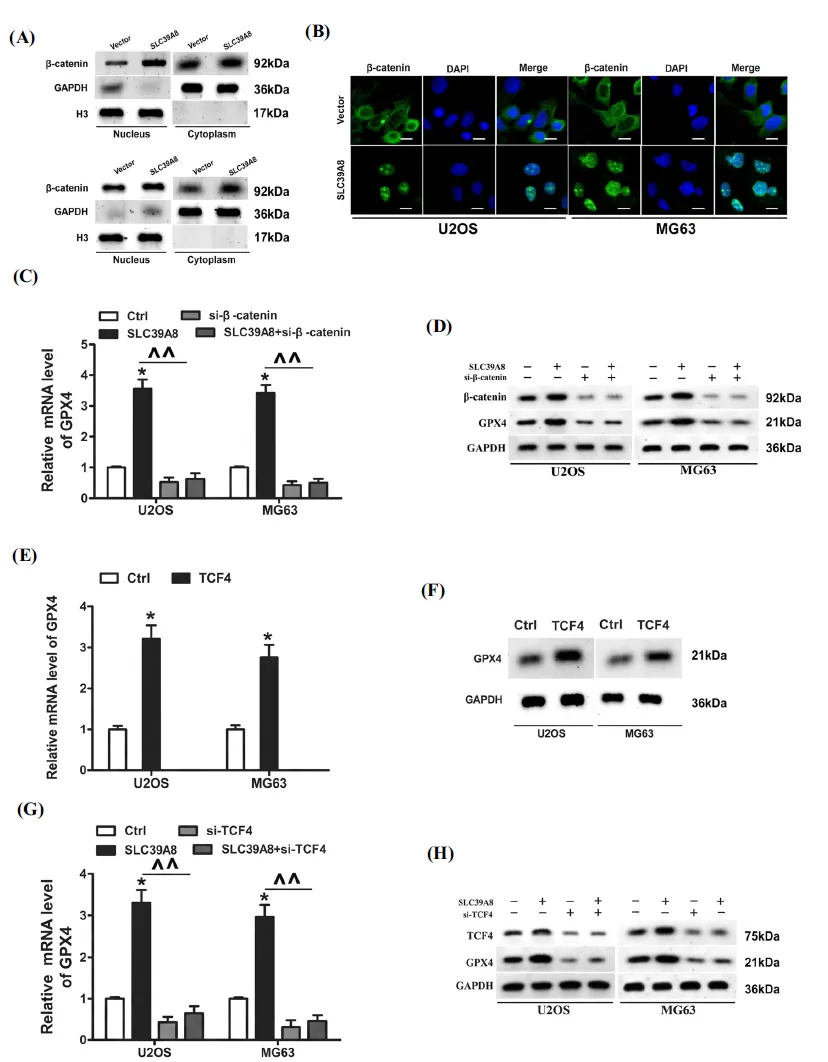
SLC39A8 increased OS cell GPX4 expression through β-catenin/TCF4. (**A**) The expression of cytoplasmic β-catenin and intranuclear β-catenin were examined by western blot. (**B**) The immunofluorescent stain of β-catenin in SLC39A8 overexpressed OS cells; scale bar: 20 μm (**C**, **D**) mRNA and protein expression of GPX4 in β-catenin-knockdown OS cells with or without SLC39A8 overexpression. (**E**, **F**) mRNA and protein expression of GPX4 in TCF4-overexpression OS cells. (**G**, **H**) mRNA and protein expression of GPX4 in TCF4-knockdown OS cells with or without SLC39A8 overexpression. Data are expressed as mean ± standard deviation. The experiments were repeated 3 times. **P <0.01 *vs.* Ctrl; ^^*P*<0.01 *vs.* sh-SLC39A8.

**Fig. (5):**
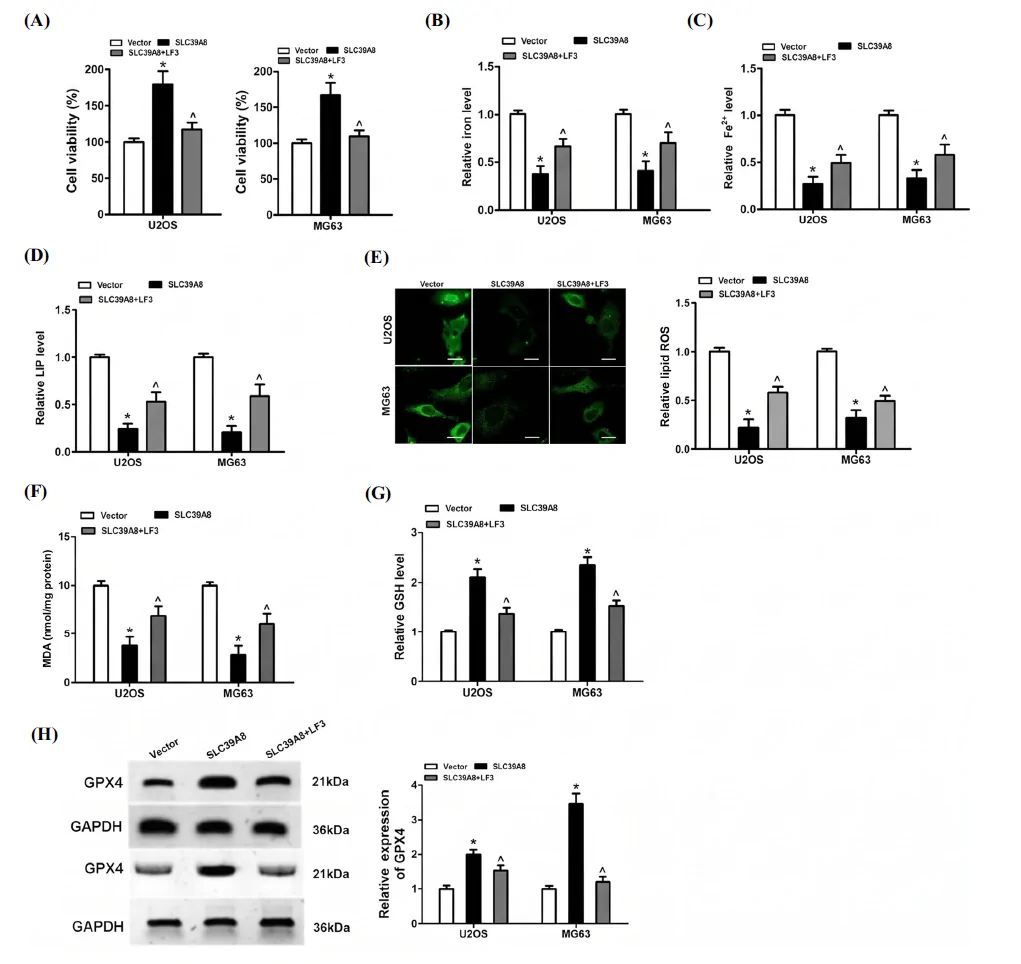
LF3 inhibited the effects of SLC39A8 on OS cells. (**A**) Cell viability was determined by CCK-8 assay. (**B**-**D**) The levels of iron, ferrous iron, and LIP were determined. (**E**-**G**) The levels of MDA, lipid ROS, and GSH were measured. Scale bar: 10 μm. (**H**) GPX4 expression was detected by western blot. Data are expressed as mean ± standard deviation. The experiments were repeated 3 times. *P <0.05 *vs.* Vector; ^*P*<0.05 *vs.* SLC39A8.

**Fig. (6):**
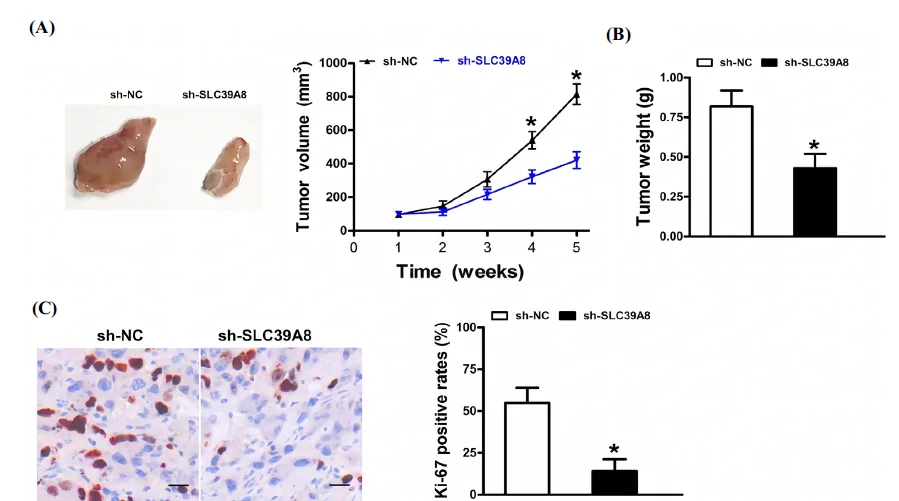
(**A**) Representative image and the volume of the tumor. (**B**) The weight of tumor xenografts. (**C**) Expression of Ki67 in xenograft tumors was detected by immunohistochemistry. Scale bar: 50 μm. *P <0.05 *vs.* sh‑NC.

## Data Availability

The datasets generated during and/or analyzed during the current study are available from the corresponding author on reasonable request.

## LIST OF ABBREVIATIONS

CCK-8: Cell Counting Kit-8
Fer-1: Ferroptosis Inhibitor-1
GPX4: Glutathione Peroxidase 4
GSH: Glutathione
LIP: Labile iron pool
MDA: Malondialdehyde
OS: Osteosarcoma
qRT-PCR: Quantitative Real-time PCR
ROS: Reactive Oxygen Species
RPE: Retinal pigment epithelium
SLC39A8: Solute carrier family 39 member 8
TCF4: Transcription factor 4
